# The Role of Porphyrin-Free-Base in the Electronic Structures and Related Properties of N-Fused Carbazole-Zinc Porphyrin Dye Sensitizers

**DOI:** 10.3390/ijms161126057

**Published:** 2015-11-19

**Authors:** Xing-Yu Li, Cai-Rong Zhang, You-Zhi Wu, Hai-Min Zhang, Wei Wang, Li-Hua Yuan, Hua Yang, Zi-Jiang Liu, Hong-Shan Chen

**Affiliations:** 1 Department of Applied Physics, Lanzhou University of Technology, Lanzhou 730050, China; lixingy@aliyun.com (X.-Y.L.); zhhm@lut.cn (H.-M.Z.); wwei@lut.cn (W.W.); yuanlh@lut.cn (L.-H.Y.); hyang@lut.cn (H.Y.); 2 State Key Laboratory of Advanced Processing and Recycling of Non-ferrous Metals, Lanzhou University of Technology, Lanzhou 730050, China; youzhiwu@lut.cn; 3 Department of Physics, Lanzhou City University, Lanzhou 730070, China; lzj@lzcu.edu.cn; 4 College of Physics and Electronic Engineering, Northwest Normal University, Lanzhou 730070, China; chenhs@nwnu.edu.cn

**Keywords:** porphyrin derivatives, dye sensitizers, electronic structures, excited state, density functional theory

## Abstract

Dye sensitizers can significantly affect power conversion efficiency of dye-sensitized solar cells (DSSCs). Porphyrin-based dyes are promising sensitizers due to their performances in DSSCs. Here, based upon a N-fused carbazole-zinc porphyrin-free-base porphyrin triad containing an ethynyl-linkage (coded as DTBC), the novel porphyrin dyes named DTBC-MP and DTBC-TP were designed by varying the porphyrin-free-base units in the π conjugation of DTBC in order to study the effect of porphyrin-free-base in the modification of electronic structures and related properties. The calculated results indicate that, the extension of the conjugate bridge with the porphyrin-free-base unit results in elevation of the highest occupied molecular orbital (HOMO) energies, decrease of the lowest unoccupied molecular orbital (LUMO) energies, reduction of the HOMO-LUMO gap, red-shift of the absorption bands, and enhancement of the absorbance. The free energy changes demonstrate that introducing more porphyrin-free-base units in the conjugate bridge induces a faster rate of electron injection. The transition properties and molecular orbital characters suggest that the different transition properties might lead to a different electron injection mechanism. In terms of electronic structure, absorption spectra, light harvesting capability, and free energy changes, the designed DTBC-TP is a promising candidate dye sensitizer for DSSCs.

## 1. Introduction

Dye-sensitized solar cells (DSSCs), which were first announced by O’Regan and Grätzel in 1991 [1,2], have attracted significant attention as promising photovoltaic devices [3,4,5]. The power conversion efficiency (PCE) can be influenced by the DSSC components, including dye sensitizers, anode/cathode, and electrolyte [4,6,7,8,9,10]. However, the vital part affecting the PCE is the dye sensitizer, performing the function of light harvesting and photon-excited electron injection [2,11,12,13,14].

During the last two decades, lots of different sensitizers such as porphyrins, metal complexes, phthalocyanines, and pure organic dyes, have been developed for DSSCs. Up to now, DSSCs based on ruthenium complexes reached a PCE over 11% [15,16,17,18], but their applications are limited by high cost, resource rareness, and environmental risk. Therefore, easy synthesis, thermal stability, and environmental friendly organic sensitizers have become the desired dye sensitizers for high PCE of DSSCs [2,3,19,20]. Among the numerous developed dye sensitizers, porphyrin dyes based on a donor-π conjugate bridge-acceptor (D-π-A) structure are promising because of their improved performance in photosynthesis, optical absorbance, and other properties [21,22,23,24]. Among them, an efficiency of 12.3% was achieved by using a D-π-A porphyrin dye YD2-*o*-C8 [25], which was comparable to the best ruthenium dye [26]. After that, a PCE up to 13% was reported from a benzothiadiazole functionalized porphyrin dye [27].

The absorption and electrochemical properties of dye sensitizers can be tuned through molecular structure modification [28,29]. It was also demonstrated that the electron injection and recombination kinetics in DSSCs can be influenced by molecular structure [30]. Considering that a large part of the solar photon energy is in the near-infrared (NIR) region, the absorption ranges and intensities of porphyrin dye sensitizers are still insufficient. Thus, it is necessary to further improve light-harvesting abilities, shift absorption bands to the NIR region, broaden absorption bands, and increase extinction coefficients in order to approach a high PCE of DSSCs. For porphyrin dye sensitizers, the four *meso* and eight β active positions can be utilized for modifying their physical, chemical and photovoltaic properties [24,31]. For instance, lots of moieties based upon triphenylamine and trimethoxyphenyl were introduced into the porphyrin *meso*-positions as electron donors, and then the effects on electronic-structure modulation and the PCE of DSSCs were further investigated [32]. Also, the designed porphyrin dyes bearing two phenyl groups at *meso*-positions of the porphine-ring with two *ortho*-substituted long alkoxyl chains exhibited significantly enhanced photovoltaic performances in DSSCs [33].

**Figure 1:**
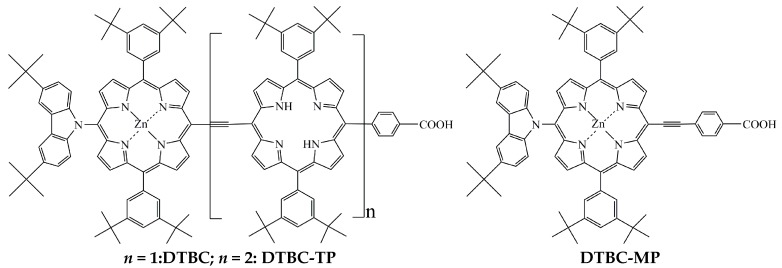
The molecular structures of porphyrin dyes DTBC, DTBC-MP, and DTBC-TP.

One way to improve optical absorption properties would be to extend the π conjugate bridge by increasing the porphyrin units. It was found that the employment of directly linked porphyrin dimers enhanced the optical, photophysical, and electrochemical performance [34]. In this avenue, a number of directly linked porphyrin arrays with different architectures (such as linear, gridlike, cyclic, *etc.*) were developed for tuning electronic and excitonic interactions [35]. The coupling of two porphyrin macrocycles through an ethynyl linker extended the porphyrin optical absorbance toward the NIR region, and their spectra for incident PCE of the diporphyrin dyes were expanded to 850 nm [36]. It was also found that the intense Soret band absorptions were broadened with the increasing of porphyrin units, and among porphyrin oligomers, ethynyl-linked porphyrin oligomers were promising due to the effective π-conjugation through rigid ethynyl linkers [37]. The reported porphyrin dimer dye sensitizer DTBC (3,6-di-*tert*-butylcarbazol-9-yl) contains the carbazole-fused zinc porphyrin coupled with free-base porphyrin. The molecular structure is given in Figure 1. DTBC was applied to fabricate DSSC with layer-by-layer architecture, where the photoanode is a nanostructured TiO_2_ film. Its efficient sensitization as far as 900 nm was found, and a PCE of 5.21% was achieved [38].

Considering the effectiveness of porphyrin-free-base in the improvement of optical absorption and the enhancement of light harvesting abilities, there are several fundamental questions to be investigated. For instance, what is the role of the number of porphyrin units in the modification of electronic structures, and how does it thus affect the optical absorption properties? What are the reasons for different performance in DSSCs with porphyrin dye sensitizers coupled to several porphyrin units? And how does the number of porphyrin units in dye sensitizers influence the dynamics of photo-induced electron injection and dye regeneration, as well as the charge transfer mechanisms? To answer these questions, based upon DTBC, we designed porphyrin dye sensitizers by changing the number of porphyrin-free-base units, which were coded as DTBC-MP and DTBC-TP. The molecular structures are also shown in Figure 1. In terms of density functional theory (DFT) and time dependent DFT (TDDFT) calculations, the effects of varying the porphyrin-free-base units in the π conjugation in electronic-structures and the related properties were studied.

## 2. Results and Discussion

### 2.1. The Geometrical Structures

**Figure 2:**
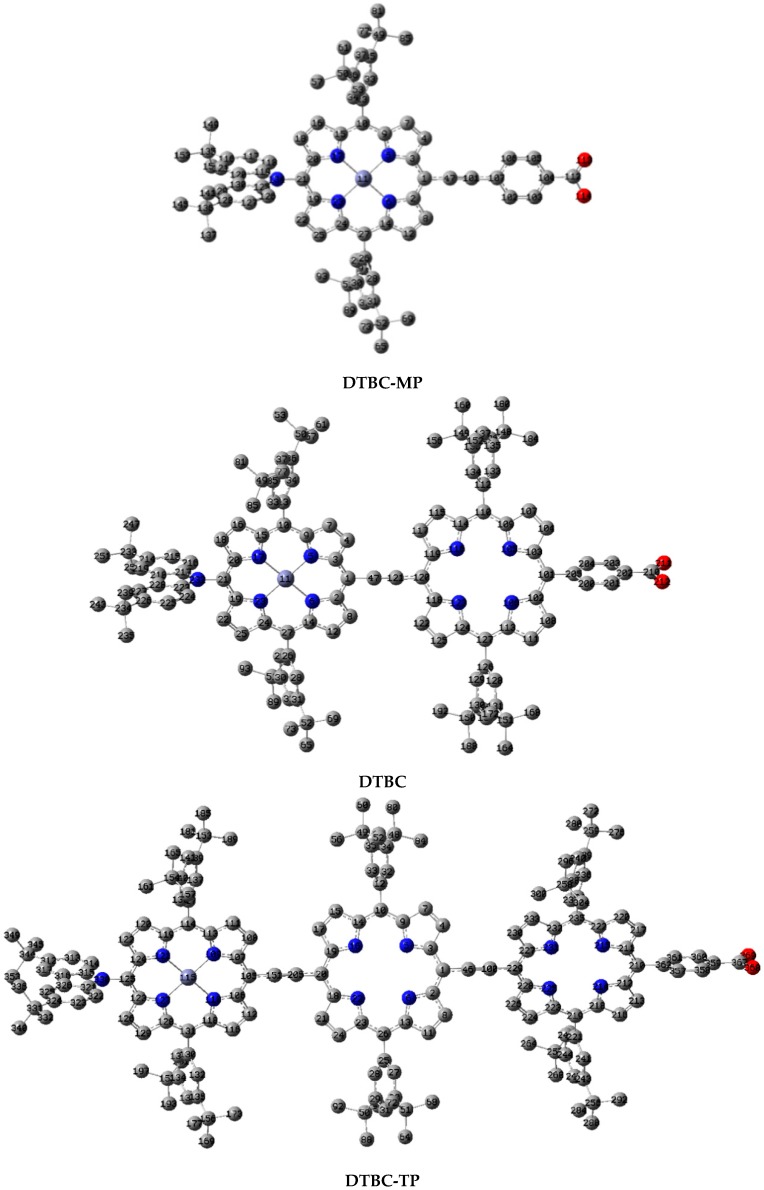
The optimized geometrical structures of DTBC-MP, DTBC, and DTBC-TP (Hydrogen atoms have been omitted for clarity, gray circles: C; blue circles: N; red circles: O; light blue circles: Zn).

In terms of the crossing or quasi-parallel orientation of the two phenyls in 3,5-di-*tert*-butylphenyl (DTBP) moieties at the *meso*-positions of porphyrin, there are several isomers for DTBC-MP, DTBC, and DTBC-TP (see Figure S1).The calculated total energies suggest that the crossing orientation of two DTBP in the same porphyrin-ring and the quasi-parallel orientation of two DTBP between two adjacent porphyrin-rings are energetically favorable. The optimized geometries for the lowest energy isomers of DTBC-MP, DTBC, and DTBC-TP are shown in Figure 2. The selected geometrical parameters are listed in Tables S1–S3. The geometrical data indicate that the variations of corresponding geometrical parameters are very tiny among DTBC-MP, DTBC, and DTBC-TP dyes. For example, for the C–N bond which connects N in carbazole and porphyrin, the bond lengths are about 0.142 nm for DTBC-MP, DTBC, DTBC-TP, and the bond angles of N–Zn–N are about 90.5°. These are reasonable due to the local character of chemical bonds. The torsion angles between carbazole and porphyrin are about 95°, 80°, and 97° for DTBC-MP, DTBC, and DTBC-TP, respectively. The torsion angle between porphyrin and Zinc-porphyrin in DTBC is about 146°, and the corresponding value in DTBC-TP is about 148°. The data of torsion angles support that, with the increasing of the porphyrin units, the torsion angle between porphyrin and zinc-porphyrin increases. This is induced by steric hindrance. Also, the torsion between porphyrin units is favorable for the reduction of dye aggregation and the inhibition of the approach of electrolyte to the semiconductor in the electrode. The introduction of rigid ethynyl in the conjugate bridges guarantees the existence of a molecular axis, which is helpful to generate a larger dipole moment and a suitable molecular orientation when dye sensitizers are adsorbed on the surfaces of the semiconductor in the electrode [39,40], and further affect the conduction band edge of the electrode materials [41].

The conjugate bridge in dye sensitizers can describe conjugate length (CL) and charge transfer (CT) distance to some extent [42,43]. In this work, the CL of dyes is defined as the distance between the N atom in the carbazole and the C atom in the COOH group. The calculated CLs are 16.60, 24.97, and 35.88 Å for DTBC-MP, DTBC, and DTBC-TP, respectively. The CL of DTBC and DTBC-TP are longer than that of YD2-*o*-C8 (about 16.7 Å) [44].

### 2.2. Electronic Structures

**Figure 3:**
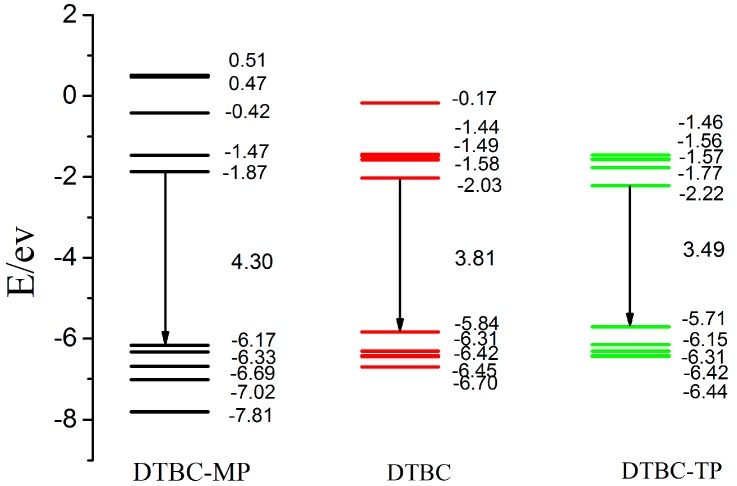
The calculated eigenvalues of frontier molecular orbitals and HOMO-LUMO gaps for DTBC-MP, DTBC, and DTBC-TP in chloroform (CAM-B3LYP/6-31g(d,p)).

The highest occupied molecular orbital (HOMO), the lowest unoccupied molecular orbital (LUMO), and HOMO-LUMO gap (HLG) of DTBC-MP, DTBC, and DTBC-TP are marked in Figure 3. The smaller HLGs of dye sensitizers are beneficial for light absorption at longer wavelength region. The calculated HOMO, LUMO, and HLG for DTBC are about −5.84, −2.03, and 3.81 eV, respectively. The increasing of porphyrin-free-base in π conjugation, corresponding to DTBC-TP, generates HOMO, LUMO, and HLG of about −5.71, −2.22, and 3.49 eV, respectively. However, the subtraction of porphyrin-free-base from DTBC, corresponding to DTBC-MP, gives HOMO, LUMO, and HLG of about −6.17, −1.87, and 4.30 eV, respectively. Apparently, porphyrin-free-base significantly affects the electronic structures of the dyes. The extension of π conjugation by using porphyrin-free-base enhances the electron delocalization, and thus lowers the LUMO, resulting in the reduction of HLG. Previous works suggest that a smaller HLG of the dye sensitizer is favorable for PCE improvement [45].The smallest HLG of DTBC-TP might correspond to better performance in DSSC. Also, theoretical works for dye sensitizers, including organic dyes [42,46,47] and Ru-complexes [48,49], indicate that the dense distribution of MO eigenvalues are favorable for the improvement of the short-circuit current density *J_sc_*. Due to the denser distribution of MO eigenvalues for DTBC-TP, the corresponding *J_sc_* might be largest if it could be applied to fabricate DSSCs.

### 2.3. Absorption Properties

**Figure 4:**
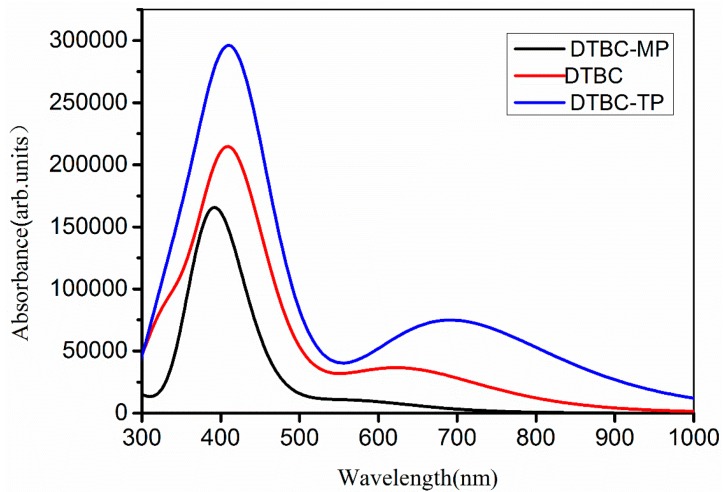
The simulated absorption spectra for DTBC-MP, DTBC and DTBC-TP based on time dependent density functional theory (TDDFT) calculated results with CAM-B3LYP/6-31G(d,p). The 0.333 eV of half-width at half-maximum was applied for absorption spectra simulations.

The simulated absorption spectra of DTBC, DTBC-MP, and DTBC-TP are given in Figure 4. The comparison of these absorption spectra indicates that, based upon DTBC, increasing porphyrin-free-base into π conjugation generates the red-shift of Q/B bands (the absorption peaks near 420 nm were called B bands, and the 500~750 nm range of absorptions were coded as Q bands) and the enhancement of absorption coefficient; however, subtraction of porphyrin-free-base from DTBC induces blue-shift of Q/B bands, and a reduction of absorption coefficient. Usually, the combination of increasing π-conjugation and decreasing molecular symmetry causes a red-shift in the molecular absorption spectrum [50]. Compared with that of DTBC, the red-shift of Q/B absorption bands of DTBC-TP results from smaller HLG and denser distribution of MO eigenvalues. The absorption strength is mainly determined by oscillator strength *f_m_*, which is computed as

(1) $$f_{m}=\frac{2m_{e}}{3{ћ}^{2}}\left(\varepsilon_{m}-\varepsilon_{k}\right){\left|r_{m,k}\right|}^{2}$$

where *m_e_* is the mass of electron, *ћ* is the reduced Planck constant, ε*_m_*(ε*_k_*) corresponds to the *m*th (*k*th) electronic levels, and |*r_m,k_*|, is the transition dipole moment between states *m* and *k* which are involved in the transition [51]. The enhancement of absorption properties of DTBC-TP relative to DTBC can be attributed to increasing |*r_m,k_*|, which is induced by the extension of π conjugation with porphyrin-free-base. Based upon absorbance and λ_max_, the absorption properties of DTBC-TP are better than those of DTBC and DTBC-MP for the application in DSSCs.

### 2.4. Excited-State Properties

To evaluate absorption properties quantitatively, one of the important physical quantities is the Einstein absorption coefficient |*B_m,k_*|. Based upon dipole approximation, |*B_m,k_*| can be obtained through perturbation theory,

(2) $$B_{m,k}=\frac{4\pi^{2}e_{s}^{2}}{3{ћ}^{2}}{\left|r_{m,k}\right|}^{2}$$

where |*B_m,k_*| is the Einstein absorption coefficient between *m* and *k* states, *ћ* is the reduced Planck’s constant, *e*_s_ = *e* (4πε_0_)^−1/2^, *e* is the elementary charge, ε*_0_* is the vacuum permittivity, and |*r_m,k_*| is the transition dipole moment from the state *m*th to *k*th. In order to give an intuitional impression for the porphyrin free base effects, we define the relative absorption coefficient |*B_m,k_*| as a ratio

(3) $$B{\left(r\right)}_{m,k}=\frac{B\left(\text{dye, }\lambda \right)}{B\text{(DTBC, 427 nm)}}$$

where *B*(dye, *λ*) is the absorption coefficient of the selected dye with the corresponding excitation at λ, and the *B* (DTBC, 427 nm) is the absorption coefficient of DTBC at about 427 nm. On the other hand, the light harvesting efficiency (LHE) is also an important factor which can affect PCE. If the excited processes of dye sensitizers have effective CT character, the dye’s LHE should be as high as possible to improve the PCE. The LHE can be calculated from LHE = 1 − 10^−ƒ^, where the ƒ is oscillator strength [52]. Table 1 lists the calculated λ_max_ (in nm), excitation energies ∆*E_m,k_* (in eV), LHE, transition dipole moments |*r_m,k_*| (in a.u.), and relative absorption coefficient *B*(*r*)*_m,k_* of Q and B bands for DTBC-MP, DTBC and DTBC-TP dyes, respectively. Apparently, for a given dye, the LHE and *B*(*r*)*_m,k_* of B band are larger than those of Q band, and for the selected absorption band, the LHE and *B*(*r*)*_m,k_* increase with the increase of the porphyrin-free-base units in the conjugate bridge. These result from increasing *r_m,k_*, which is generated from the enhancement of electronic delocalization due to the increase in the number of porphyrin-free-base units.

**Table 1:** The calculated light harvesting efficiencies (LHE), transition dipole moments (*r_m,k_*, in a.u.) and relative absorption coefficients *B*(*r*)*_m,k_* of Q and B bands (the absorption peaks near 420 nm were called B bands , and the 500~750 nm range of absorptions were coded as Q bands) for DTBC-MP, DTBC and DTBC-TP dyes. The corresponding λ_max_ (in nm) and excitation energies ∆*E_m,k_* (in eV) are also listed.

Dyes	λ_max_	∆*E_m,k_*	*r_m,k_*	*B*(*r*)*_m,k_*	LHE
DTBC-MP	565	2.19	4.47	0.01	0.425
	387	3.21	22.60	0.35	0.983
DTBC	637	1.95	15.77	0.17	0.823
	427	2.90	38.14	1	0.998
DTBC-TP	699	1.77	40.06	1.10	0.982
	435	2.85	43.99	1.33	0.999

The calculated excitation energies (eV), excitation wavelength (nm), oscillator strengths (*f*) and major transition configurations with coefficients larger than 10% are listed in Table 2. The related MOs are displayed in Figure S2. It can be found from the MO analysis that the *tert*-butyl moieties of DTBC-MP, DTBC, DTBC-TP are independent of their frontier MOs. This suggests that the *tert*-butyl groups play the role of reducing dye aggregation [53], blocking the approach of electrolyte to the semiconductor electrode surfaces, and suppressing charge recombination [39,44]. For DTBC-MP, the Q and B band transitions that involved H → L, H − 2 → L, H − 1 → L (the H and L stand for HOMO and LUMO, respectively) configurations are intra-molecular CT excitations due to the relocation of MOs between the ground and final states. Since the LUMO of DTBC-MP extends to the carboxyl moiety which is an anchor group, the transitions at λ_max_ of the Q and B bands are effective CT excitation which is favorable for photon-induced electron injection in DSSC [46]. While the transitions composed of H − 1 → L + 1 and H → L + 1 configurations are mainly local excitation in porphyrin. For DTBC, the carbazole moiety is not an effective electronic donor since it does not contribute to the frontier occupied MOs. The absorption at about 637 nm is mainly contributed from the H → L configuration. The HOMO and LUMO are distributed in the π system of zinc porphyrin-free-base porphyrin with an ethynyl-linkage so the transition exhibits local excitation character. Meanwhile, the absorption at about 427 nm is also local excitation due to localized transition configurations which mainly relate to zinc-porphyrin/porphyrin groups. However, the absorption at about 397 nm has intra-molecular CT character to some extent because of the relocations of MOs in transition configurations. Furthermore, the smaller contribution of the anchoring group to the virtual orbitals involved in transitions is one of the reasons why the PCE is only 5.21% [46]. For DTBC-TP, the role of the carbazole moiety is similar to that in DTBC. The absorption at about 699 nm is mainly contributed from the H → L configuration, and it is local excitation since a similar distribution of HOMO and LUMO extend the π system of the zinc porphyrin and porphyrin-rings. The transition configurations at about 435 nm also exhibit local excitation character because the related MOs are mainly distributed in different porphyrin units, whereas, the absorption at about 402 nm shows intra-molecular CT character due to the MOs relocations between the initial and final states. Similar to the case of DTBC, the contribution of the anchoring group to virtual MOs involved in transitions is also tiny.

The analysis of transition properties and MOs also indicates the following: increases the number of porphyrin free-base units in the conjugate bridge, promotes the overlap between the ground and excited states, so that the absorption is enhanced. Also, the MOs related to the transition in the UV-vis region become more delocalized with the increase in the number of porphyrin free-base units, which further induces the red-shift of the absorption bands.

The analysis of the transition properties could help us to understand the electron injection mechanism. Based upon the study of model systems for zinc porphyrin dye-sensitized solar cells, three possible pathways of electron injection from the excited dyes to the TiO_2_ nanoparticles have been presented [54]: the first mode is the fastest direct one-step injection by photo-excitation. The second injection pathway is that, when dye sensitizers are excited, some of the excited electrons are pumped to an anchoring moiety, and then transfer from the ligand to the surface of the electrode semiconductor. This is the most effective and fast electron injection mode and should be a nonadiabatic and nonradiation process. The third pathway with the longer time scale injects electrons from the excited porphyrin dye sensitizers to the electrode semiconductor. Through the analysis of transition configurations and MOs, the main electron injection mode of DTBC-MP might be through the first or second pathways because the excitation pumps electrons to the anchor group. However, the major electron injection pathways of DTBC and DTBC-TP are the third pathway of the above discussed since the excitation of the Q/B bands are mainly local excitation of porphyrins. As a result, different porphyrin free base units in the conjugate bridge may lead to different electron injection mechanisms.

**Table 2:** The calculated excitation energies (eV), excitation wavelength (nm), oscillator strengths (*f*) and major transition configurations with coefficients larger than 10% of DTBC-MP, DTBC, and DTBC-TP in chloroform solution (CAM-B3LYP/6-31G(d,p)).

Dye	States	Major Transition Configurations	λ (eV/nm)	*f*
DTBC-MP	S_1_	(69%) H → L; (29%) H − 1 → L + 1	2.19/565	0.2405
	S_3_	(50%) H − 2 → L; (28%) H − 1 → L + 1; (14%) H → L	3.11/399	0.9522
	S_4_	(40%) H − 2 → L; (28%) H − 1 → L + 1; (14%) H → L	3.14/395	1.3916
	S_5_	(59%) H → L + 1; (41%) H − 1 → L	3.21/387	1.7749
DTBC	S_1_	(66%) H → L	1.95/637	0.7521
	S_2_	(32%) H → L + 1; (26%) H − 2 → L	2.12/584	0.1576
	S_5_	(36%) H − 1→ L + 3; (28%) H − 2→ L + 1; (18%) H → L	2.90/427	2.7116
	S_6_	(27%) H → L + 1; (24%) H − 2 → L; (17%) H → L + 3; (11%) H − 1 → L	3.06/405	0.2974
	S_7_	(31%) H → L + 3; (28%) H − 1 → L; (15%) H → L + 1; (13%) H – 2 → L	3.12/397	2.4995
DTBC-TP	S_1_	(73%) H → L	1.77/699	1.7398
	S_3_	(24%) H → L + 3; (20%) H − 4 → L	2.08/596	0.1285
	S_7_	(39%) H − 4 → L + 3; (17%) H − 3 → L + 2; (12%) H → L; (10%) H − 2 → L + 5	2.86/435	3.0730
	S_8_	(54%) H – 1 → L; (13%) H → L + 1; (12%) H − 2 → L + 5	2.89/429	0.4341
	S_10_	(41%) H − 4 → L; (37%) H → L + 3	2.98/415	0.5251
	S_11_	(23%) H – 3 → L; (23%) H → L + 2; (12%) H − 2 → L; (11%) H − 3→ L + 1; (11%) H → L + 5	3.07/404	0.1376
	S_13_	(25%) H – 2 → L; (20%) H → L + 5; (13%) H → L + 2; (12%) H − 3 → L	3.09/402	2.9599
	S_15_	(23%) H − 3→ L + 2; (14%) H − 4 → L + 3; (11%) H − 2 → L + 5; (21%) H − 1→ L + 1	3.38/367	0.6364

### 2.5.The Free Energy Variation of Electron Injection and Dye Regeneration

Generally, the electron injection from the excited dyes to the conduction band of the semiconductor electrode and the dye regeneration process can be viewed as a CT reaction. In terms of Marcus theory of CT [55], the free energy variation for electron injection (∆*G^inject^*) and dye regeneration (∆*G^regen^*) can affect the rate of photon-excited electron injection and dye regeneration, respectively, and then influence short-circuit current density *J_sc_* and open-circuit voltage *V_oc_*.

Table 3 lists the calculated values of the ground state oxidation potential (GSOP) $E_{OX}^{dye}$, the excited state oxidation potential (ESOP) $E_{OX}^{dye*}$, ∆*G^inject^* and ∆*G^regen^* for DTBC-MP, DTBC, DTBC-TP. The data of $E_{OX}^{dye}$ and $E_{OX}^{dye*}$ indicate that the extension of the conjugate bridge using porphyrin free base reduces the GSOP and ESOP. This results from the pophyrin free base effects on the shift of HOMO and λ_max_. The negative values of ∆*G^inject^* mean the dye’s excited-states lie above the electrode semiconductor conduction band edge, providing driving force for electron injection from the photo-excited sensitizers to the conduction band of the electrode semiconductor. This ensures an effective electron injection that can occur at the interface between dye sensitizers and electrode in DSSCs. Furthermore, the absolute values of ∆*G^inject^* for Q bands are smaller than that of B bands. The tendency agrees with that of other porphyrin dyes, such as YD2-*o*-C8 [39,44]. This suggests that the rate of electron injection from B bands might be faster than that from Q bands. Meanwhile, the absolute values of ∆*G^inject^* for B bands increase with increasing porphyrin-free-base units. This indicates that introducing more porphyrin-free-base in the conjugate bridge induces a faster rate of electron injection, which is favorable to improve *J_sc_* and *V_oc_*. Considering the absorption of B bands are stronger than that of Q bands, the overall rate of electron injection for DTBC-TP could be the fastest among the dyes investigated.

The dye regeneration rates are sensitive to reaction driving forces [56]. Meanwhile, the recombination between electrons in the semiconductor electrode and dye cations depends upon the kinetic competition between the electron back flow itself and the dye regeneration. Given an electron back flow rate, the faster the regeneration, the less it is that the electrons can be recaptured by the oxidized dye cations. The dye regeneration requires that the GSOP must be at least ~0.2 eV lower than the redox potential of electrolyte [57]. Though the values of ∆*G^regen^* decrease with increasing porphyrin units in the conjugate bridge, they are still large enough for effective dye regeneration.

**Table 3:** The calculated oxidized potential of ground state ($E_{OX}^{dye}$) and excited-state ($E_{OX}^{dye*}$), as well as the free energy variation of electron injection (∆*G^inject^*) and dye regeneration (∆*G^regen^*) for DTBC-MP, DTBC, and DTBC-TP (all of the quantities are giving in eV).

Dyes	$E_{OX}^{dye}$	$E_{OX}^{dye*}$		∆*G^inject^*		∆*G^regen^*
		Q	B	Q	B	
DTBC-MP	6.17	3.98	2.96	−0.02	−1.04	1.32
DTBC	5.84	3.89	2.94	−0.11	−1.06	0.99
DTBC-TP	5.71	3.94	2.86	−0.06	−1.14	0.86

## 3. Computational Methods

DFT and TDDFT calculations were performed by using the Gaussian09 package [58]. The long-range corrected functional CAM-B3LYP [59,60] and the polarized split-valence basis sets 6-31g(d,p) were adopted for geometry optimization of DTBC and designed dyes. The functional CAM-B3LYP was successfully applied to investigate the electronic structures and related properties of tetrahydroquinoline dye sensitizers [43]. It was also demonstrated that 6-31G(d,p) basis sets were sufficient for calculating the excitation properties and electron density of organic dyes [61,62]. Since the absorption spectrum of DTBC was measured in chloroform solution [38], the electronic structures and related properties of DTBC, DTBC-MP, and DTBC-TP were calculated in chloroform solution in order to compare them under the same conditions. The solvent effects were considered using a polarizable continuum model (PCM) method [63,64]. The calculations proved that the solvent effects generated negligible influence on the geometry of porphyrin dyes [39]. Therefore, considering the computational cost and accuracy, the TDDFT calculations in solution for the dyes in this work were conducted on the optimized geometries in the gas phase.

Functional selection is important for the accurate computation of the dyes’ excitation properties because the TDDFT calculations with conventional hybrid functionals usually underestimate CT excitations energies, and a different functional may generate different transition properties [48]. In order to select a suitable functional for the reliable description of excited properties, TDDFT calculations were performed using the CAM-B3LYP, PBE0 [65,66,67], M062X [68], and HSE06 [69,70,71,72,73] functionals. The performance of these functionals was better than that of other functionals in the excitation calculations of porphyrin dyes [44]. Porphyrin compounds in the UV-vis region have a characteristic absorption—strong absorption peaks near 420 nm called Soret bands (B), and the 500~750 nm range of several weak absorptions for the Q zone [74]. The calculated absorption λ_max_ (nm/eV), absolute errors (AE, in eV), and arithmetic mean absolute errors (AMAE, in eV) of DTBC are listed in Table 4. The data indicates that the suitable functionals for B and Q bands are CAM-B3LYP and HSE06, respectively. On average, the CAM-B3LYP functional generated the smallest AMAE for these bands (about 0.255 eV). Therefore, the CAM-B3LYP functional was adopted in TDDFT calculations for excitation analysis of DTBC, DTBC-MP, and DTBC-TP.

**Table 4:** The calculated absorption λ_max_ (in nm/eV), the absolute errors (AE, in nm/eV), and the arithmetic mean absolute errors (AMAE, in eV) of B and Q bands for DTBC with different fuctionals in TDDFT.

	CAM-B3LYP		HSE06		M062X		PBE0		Experiment	
	B	Q	B	Q	B	Q	B	Q	B	Q
λ_max_	2.90/427	1.95/637	3.14/395	1.81/685	2.91/427	1.99/622	3.21/386	1.88/660	2.61/478	1.73/722
AE	0.29	0.22	0.53	0.08	0.30	0.26	0.60	0.15		
AMAE	0.255		0.305		0.280		0.375			

The free energy variation for electron injection ∆*G^inject^* affects the electron injection rate and thus influences the short circuit current density *J_sc_*. In terms of Preat’s method [52], the ∆*G^inject^* can be calculated from the formula $\Delta G^{inject}=E_{OX}^{dye*}-E_{CB}$, where $E_{OX}^{dye\text{*}}$ is the ESOP of the dye and *E_CB_* is the reduction potential of the conduction band of the semiconductor. Apparently, the ∆*G^inject^* linearly depends on the $E_{OX}^{dye\text{*}}$, and *E_CB_* is a parameter which corresponds to the semiconductor applied in the electrode. The commonly used semiconductor material for DSSC’s electrode is anatase TiO_2_. So, the reported *E_CB_* for anatase TiO_2_ (about 4.0 eV) [75] was adopted in this work as reference value. The $E_{OX}^{dye\text{*}}$ can be calculated as $E_{OX}^{dye*}=E_{OX}^{dye}-\lambda_{\max}$ [76], in which $E_{OX}^{dye}$ is the GSOP and λ_max_ is the absorption maximum, whereas the free energy variation for dye regeneration ∆*G^regen^* can affect the rate constant of the redox process between the oxidized dyes and electrolyte. The ∆*G^regen^* can be calculated as $\Delta G^{regen}=E_{OX}^{dye}-E_{redox}^{electrolyte}$, where $E_{redox}^{electrolyte}$ is the redox potential of the electrolyte. The $E_{redox}^{electrolyte}$ of commonly used redox couple iodide/triiodide (about 4.85 eV, 0.35 V *vs.* NHE) [77] was adopted as reference to evaluate the porphyrin-free-base effects on ∆*G^regen^*.

## 4. Conclusions

In this work, in order to investigate the role of porphyrin-free-base in the modification of electronic structures and related properties, we designed the novel porphyrin dyes named DTBC-MP and DTBC-TP by varying the porphyrin-free-base units in the π conjugation of DTBC. The geometries, electronic structures, excitations, and free energy changes were calculated. The results indicates that, the extension of the conjugate bridge with porphyrin-free-base units results in elevation of the HOMO energies, decrease of the LUMO energies, reduction of HLG, red-shift of the absorption bands, and enhancement of the absorbance. Meanwhile, the free energy changes demonstrate that introducing more porphyrin-free-base in the conjugate bridge induces a faster rate of electron injection. All of the designed dyes have enough free energy variations for dye regeneration. Furthermore, the transition properties and MO characters suggest that the different transition properties lead to a different sensitization mechanism, and electron transfer occurs from the excited porphyrins to the semiconductor electrode for DTBC and DTBC-TP. In terms of electronic structures, absorption spectra, light harvesting efficiency, and free energy changes, the designed DTBC-TP is a promising candidate dye sensitizer for DSSCs.

Extensions of the present work would be to explore the position effect of the zinc-porphyrin unit in DTBC and DTBC-TP. The good electronic coupling between zinc-porphyrin and phenylcarboxylic acid through the ethynyl-linkage [38,43] is favorable for fast electron injection modes. On the other hand, adoption of more effective electron-donor groups, such as the diarylamino group with two hexyl chains in YD2-o-C8, can further improve the performance in DSSCs. However, due to computational demand, this will only be investigated in future work.

## Supplementary Materials

Supplementary materials can be found at http://www.mdpi.com/1422-0067/16/11/26057/s1.
