# Supplementary material for: The Role of Porphyrin-Free-Base in the Electronic Structures and Related Properties of N-Fused Carbazole-Zinc Porphyrin Dye Sensitizers
Source: Int J Mol Sci. 2015 Nov 19;16(11):27707–20. doi: 10.3390/ijms161126057 (PMC4661915; doi:10.3390/ijms161126057)
Supplement: Supplementary file 1 [file ijms-16-26057-s001.pdf]

Supplementary Materials: The Role of Porphyrin–Free-Base in the Electronic Structures and Related Properties of N-Fused Carbazole–Zinc Porphyrin Dye Sensitizers

Xing-Yu Li, Cai-Rong Zhang, You-Zhi Wu, Hai-Min Zhang, Wei Wang, Li-Hua Yuan, Hua Yang, Zi-Jiang Liu and Hong-Shan Chen

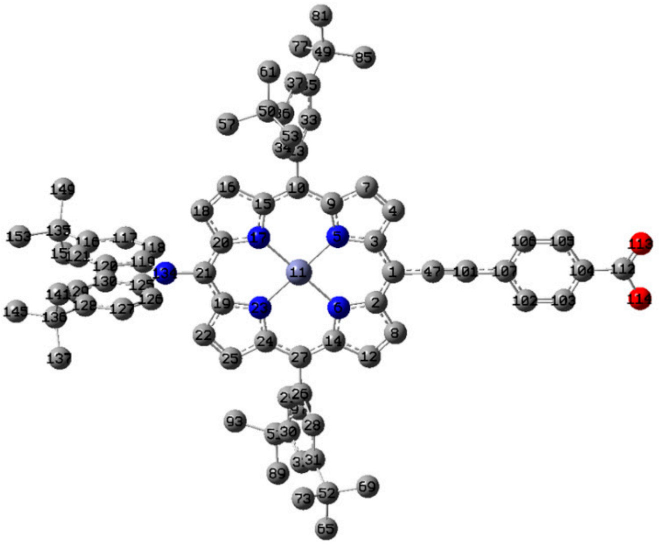

DTBC-MP

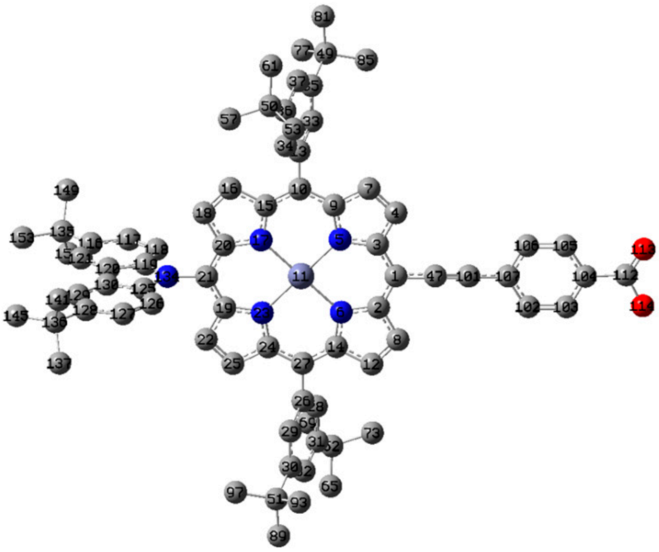

DTBC-MP-2

Figure S1. *Cont.*

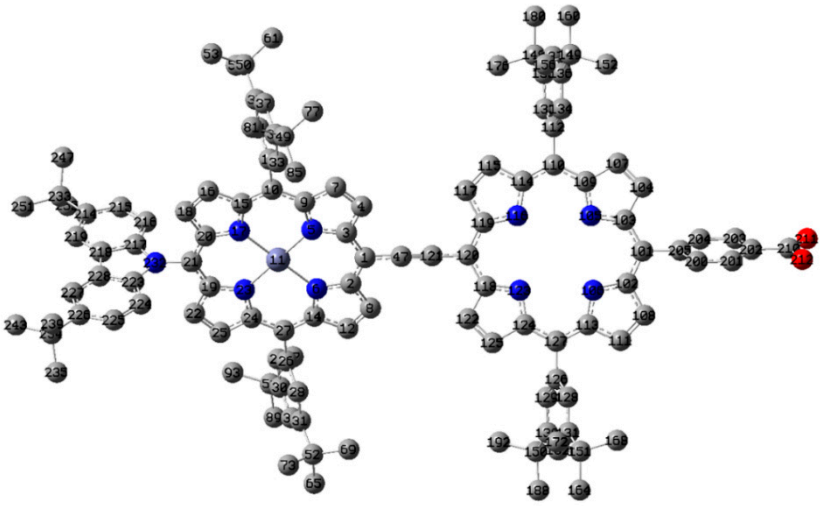

DTBC-1

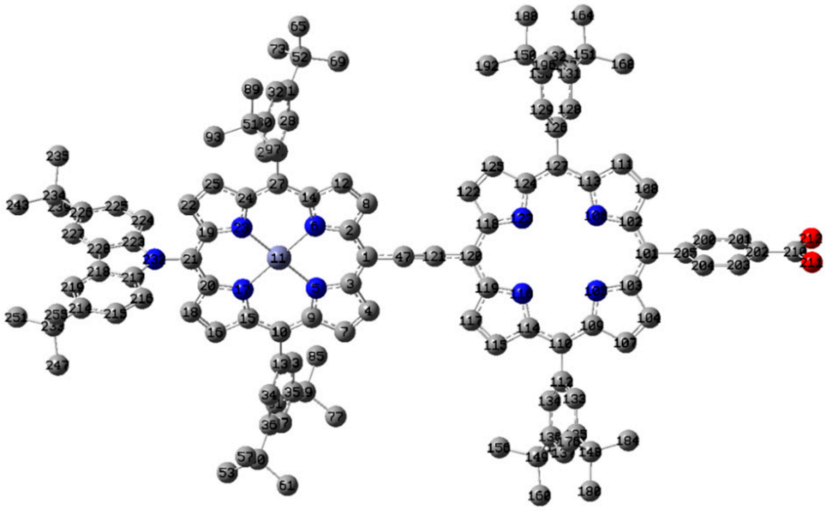

DTBC-2

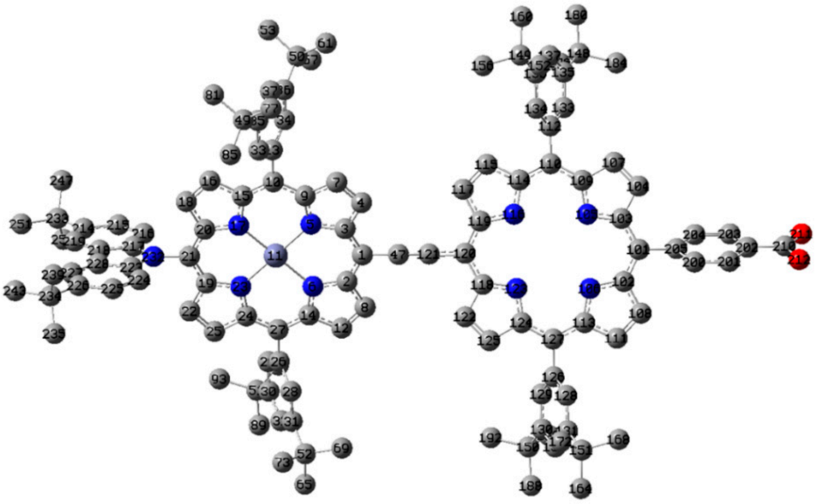

DTBC

Figure S1. *Cont.*

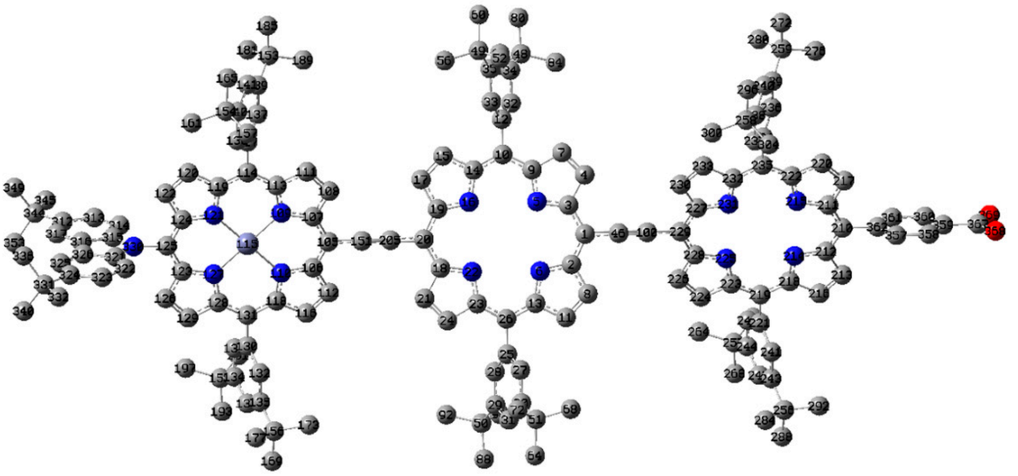

DTBC-TP

Figure S1. The optimized geometrical structures of DTBC-MP, DTBC-MP-2, DTBC-1, DTBC-2, DTBC and DTBC-TP with atomic serial numbers (Hydrogen atoms have been omitted for clarity, gray circles: C, blue circles: N, red circles: O, light blue circles: Zn).

Table S1. The selected bond lengths or distances between atom (in Å), bond angles (in degree), and dihedral angels (in degree) of DTBC-MP. Two, three, and four atomic-serial numbers represent bond length, bond angle, and dihedral angle, respectively.

Definition	Values	Definition	Values	Definition	Values
21-134	1.42	21-134-125	126.0	125-134-21-19	94.8
134-125	1.39	21-134-119	126.0	119-134-21-20	94.8
134-119	1.39	125-134-119	108.2	19-23-11-5	−92.5
6-11	2.03	5-11-6	90.5	20-17-11-6	−92.3
5-11	2.03	5-11-17	89.5	24-27-26-29	67.8
11-17	2.03	6-11-17	178.8	15-10-13-34	67.8
11-23	2.03	17-11-23	90.5	14-27-26-28	67.8
1-47	1.42	1-47-101	180.0	9-10-13-33	67.8
47-101	1.21	47-101-107	180.0	8-2-1-47	−1.6
101-107	1.43	1-101-107	180.0	4-3-1-47	−1.6
104-112	1.48	104-112-113	124.8	1-47-101-107	4.5
112-113	1.21	104-112-114	113.2	47-101-107-102	−178.8
112-114	1.35	113-112-114	122.0	47-101-107-106	1.2
10-13	1.49	10-13-33	120.4	103-104-112-114	0.0
26-27	1.49	27-26-28	120.4	105-104-112-113	0.0

Table S2. The selected bond lengths or distances between atom (in Å), bond angles (in degree), and dihedral angels (in degree) of DTBC. Two, three, and four atomic-serial numbers represent bond length, bond angle, and dihedral angle, respectively.

Definition	Values	Definition	Values	Definition	Values
21-232	1.42	21-232-223	126.0	223-232-21-19	99.8
232-223	1.39	21-232-217	126.0	217-232-21-20	99.6
232-217	1.39	223-232-217	108.1	19-23-11-5	−91.3
6-11	2.03	5-11-6	90.5	20-17-11-6	−92.3
5-11	2.03	5-11-17	89.5	24-27-26-28	67.0
11-17	2.03	6-11-17	178.9	15-10-13-33	67.4
11-23	2.03	17-11-23	90.5	14-17-26-29	67.4
123-105	4.05	105-123-116	45.6	9-10-13-34	67.4
123-116	2.93	105-123-106	46.4	8-2-1-47	−2.1

Table S2. *Cont.*

Definition	Values	Definition	Values	Definition	Values
116-106	4.20	116-106-123	44.3	4-3-1-47	−3.0
123-106	2.90	123-116-106	43.7	1-47-121-120	74.9
1-47	1.43	1-47-121	180.0	121-120-118-122	−2.6
47-121	1.21	47-121-120	178.0	121-120-119-117	−1.9
121-120	1.42	1-121-120	179.4	118-123-105-103	174.6
101-205	1.49	1-47-120	180.0	119-116-106-102	175.4
202-210	1.48	202-210-211	125.0	113-127-126-128	66.0
210-211	1.21	202-210-212	113.2	114-110-112-134	66.2
210-212	1.35	211-210-212	122.0	124-127-126-129	66.0
10-13	1.49	10-13-34	120.3	108-102-101-205	−3.5
27-26	1.49	27-26-28	120.5	104-103-101-205	−4.3
127-126	1.49	127-126-128	120.5	201-202-210-212	0.2
110-112	1.49	110-112-133	120.3	203-202-210-211	0.2

Table S3. The selected bond lengths or distances between atom (in Å), bond angles (in degree), and dihedral angels (in degree) of DTBC-TP. Two, three, and four atomic-serial numbers represent bond length, bond angle, and dihedral angle, respectively.

Definition	Values	Definition	Values	Definition	Values
125-330	1.42	125-330-315	125.9	123-125-330-321	97.0
330-315	1.39	125-330-321	125.9	124-125-330-315	97.0
330-321	1.39	315-330-321	108.2	124-121-115-110	−92.1
127-115	2.03	127-115-110	89.5	123-127-115-109	−91.3
115-110	2.03	127-115-109	178.8	118-131-130-132	67.3
115-109	2.03	110-115-109	90.5	113-114-117-137	67.1
115-121	2.03	109-115-121	89.5	133-130-131-128	67.1
5-22	4.05	6-5-22	45.4	119-114-117-138	67.0
5-6	2.95	16-5-22	45.4	112-106-105-151	−2.2
6-16	4.20	5-6-16	43.4	108-107-105-151	−2.8
5-16	2.89	6-16-5	44.6	105-151-205-20	114.2
231-214	4.05	214-231-225	46.3	21-18-20-205	−2.8
231-225	2.93	231-225-215	43.7	17-19-20-205	−2.5
225-215	4.20	231-225-214	88.0	18-22-5-3	177.0
225-214	2.90	214-225-215	44.3	19-16-6-2	177.4
105-151	1.42	151-105-205	179.6	13-16-25-27	67.2
105-205	1.21	20-105-205	179.6	9-10-12-32	66.8
105-20	1.42	20-205-151	179.6	23-26-25-28	67.5
1-46	1.43	1-46-100	179.7	33-12-10-14	67.1
46-100	1.21	46-100-229	179.7	8-2-1-46	0.2
100-229	1.43	1-100-229	179.6	4-3-1-46	−0.7
210-362	1.50	1-46-229	179.6	1-46-100-229	63.5
359-367	1.48	359-367-369	113.2	100-229-228-226	−0.4
367-369	1.35	359-367-368	124.8	100-229-227-230	−0.7
367-368	1.21	368-367-369	122.0	227-231-214-212	178.9
114-117	1.49	114-117-137	120.4	228-225-215-222	−0.7
130-131	1.49	13-130-132	120.4	218-219-221-241	69.1
10-12	1.49	10-12-32	120.4	222-235-234-236	69.3
25-26	1.49	26-25-27	120.6	211-210-362-361	−110.0
219-221	1.50	219-221-241	120.4	358-359-367-368	-0.2
234-235	1.50	235-234-237	120.4	360-359-367-369	−0.2

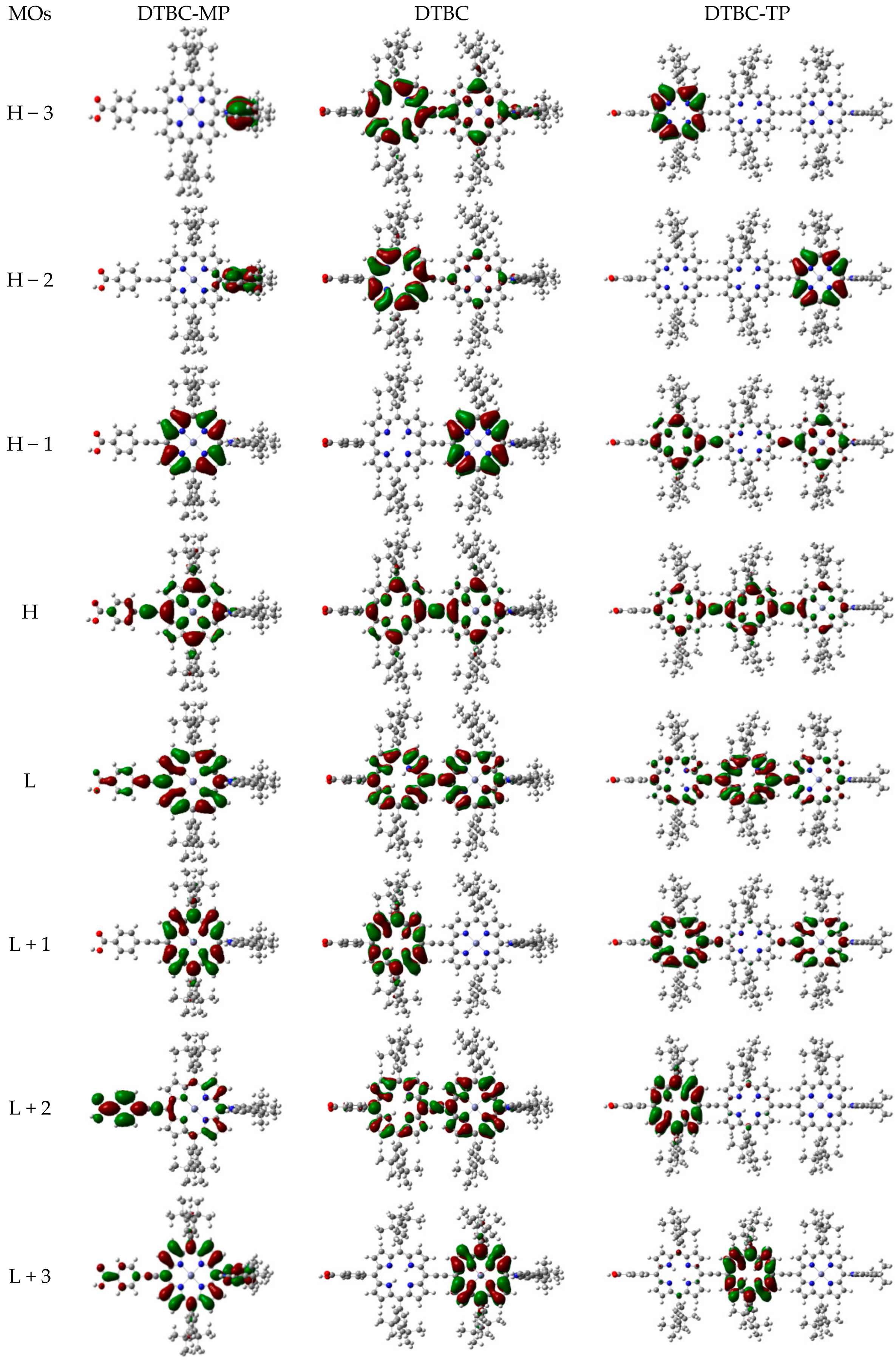

Figure S2. Isodensity plots (isodensity contour = 0.02 a.u.) of the frontier orbitalsof the dyes DTBC-MP, DTBC, and DTBC-TP. (H = HOMO, L = LUMO). Different colors stand for α and β spin, respectively.
